# Two cases of unilateral limbal Vernal keratoconjunctivitis in the same family: first case report

**DOI:** 10.3389/fped.2023.1237760

**Published:** 2023-10-02

**Authors:** Paola Michieletto, Attilio Sica, Egidio Barbi, Stefano Pensiero

**Affiliations:** ^1^ Institute for Maternal and Child Health - IRCCS “Burlo Garofolo”, Trieste, Italy; ^2^ University of Trieste, Trieste, Italy

**Keywords:** unilateral limbal Vernal keratoconjunctivitis (VKC), pediatric ophthalmology, first report, IgE-mediated hypersensitivity, allergic asthma

## Abstract

This case report describes two cases of unilateral limbal Vernal keratoconjunctivitis (VKC) in the same family. To our knowledge, these are the first two reported cases of unilateral limbal VKC. VKC is a chronic inflammatory disease that typically affects both eyes, with unilateral cases being rare and previously only reported in the tarsal form. Our first case involved a 12-year-old girl with a history of allergic asthma, who had been experiencing conjunctivitis in her right eye since the age of 7. Upon examination, she was diagnosed with unilateral limbal VKC and treated with 1% cyclosporine eye drops with a significant improvement observed at the one and three-month follow-ups. Her 7-year-old brother was also examined and found to have unilateral limbal VKC in his right eye, although it was milder and not associated with allergic pathogenesis. Therefore, in this case, a treatment with hydrocortisone eye drops was started leading to an immediate reduction of the itching. In both cases an IgE-mediated mechanism is less likely because of the monolateral eye involvement, the complete absence of nasal symptoms, the lack of correlation between symptoms and any pollen season, and the negative prick skin test in one of the two siblings. Both cases suggest that unilateral VKC may occur even in the limbal form and that genetic mechanisms may contribute to the inflammatory reaction in VKC. This report highlights the need for further studies to explain the occurrence of unilateral VKC cases and reminds clinicians to consider the possibility of unilateral limbal VKC in pediatric patients.

## Introduction

Vernal keratoconjunctivitis (VKC) is a chronic seasonal inflammatory disease with a prevalence of <1/10,000 in Europe (1). It is more commonly found in males and typically starts at around 6–7 years of age. VKC almost always affects both eyes (2) and can be diagnosed based on its clinical features (3). There are three different clinical forms of VKC: the tarsal form, which is characterized by a giant papillary reaction of the upper tarsal conjunctiva; the limbal form, which is characterized by a gelatinous infiltration around the cornea; and the mixed form, which exhibits both tarsal and limbal papillary reactions (4).

Cases of unilateral VKC are rare and have previously only been reported in the tarsal form (2, 5–7). Nevertheless, we have documented two cases of unilateral VKC, both limbal, which were found in members of the same family.

## Case description

We observed a 12-year-old girl who had been experiencing conjunctivitis in her right eye since the age of 7. Her symptoms included redness, excessive tearing, itching, and sensitivity to light. Her condition worsened during the summer months and was treated with repeated cycles of tobramycin/dexamethasone eye drops in the right eye only. This treatment provided relief from the symptoms, especially the itching, but they resumed shortly after its suspension. Therefore, tobramycin/dexamethasone eye drops were administered several times over the summer, each time for a duration of 7–10 days. The girl also had a history of allergic asthma, which her mother also suffered from. There was no history of allergic rhinitis nor concurrent nasal symptoms, which are typically present in IgE-mediated conjunctivitis. Furthermore, the onset of conjunctivitis was not aligned with any typical pollen season. The prick test results showed that she was sensitized to dust mites, birch trees, mugwort, oleaceae, and cat hair.

**Figure 1:**
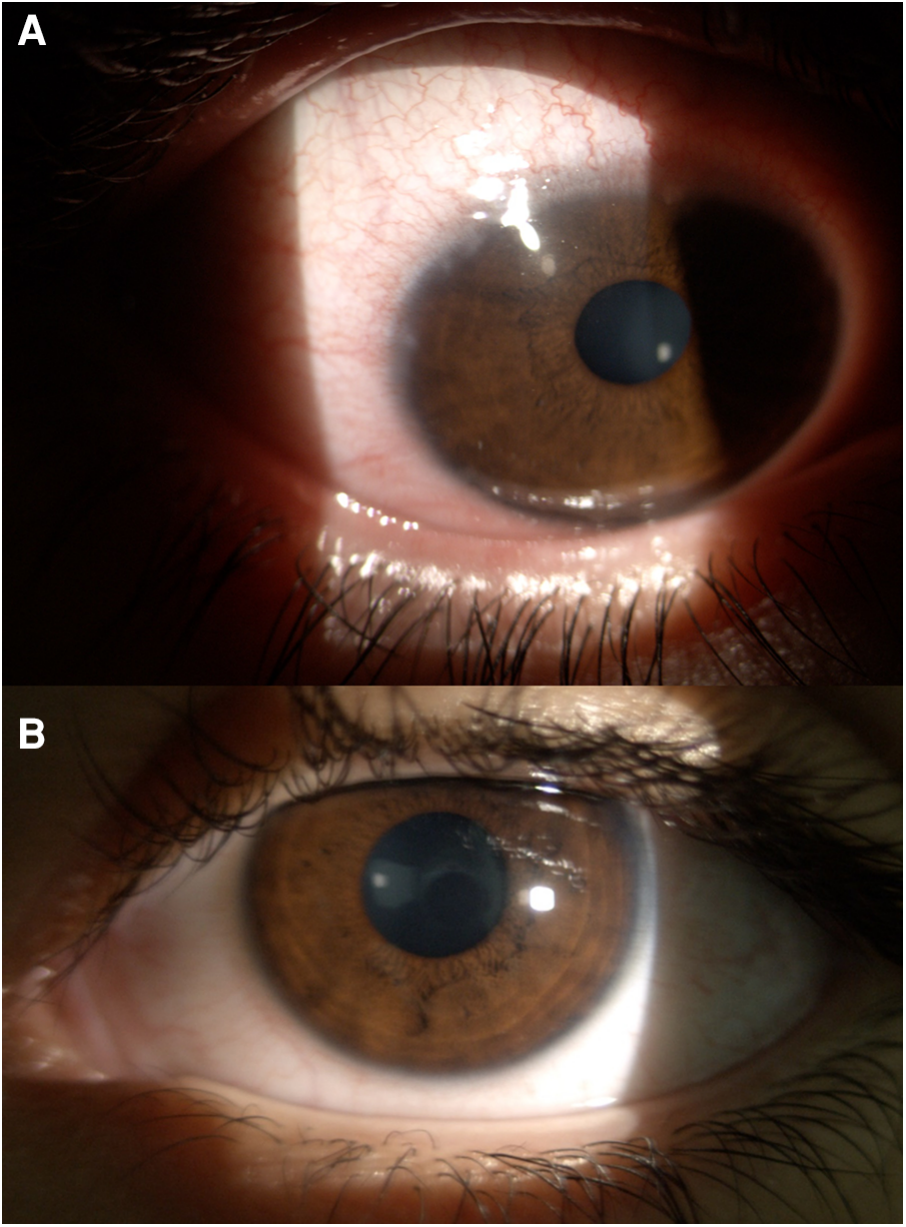
Slit-lamp examination of a 12-year-old girl's right eye (**A**, upper panel) reveals unilateral limbal VKC, characterized by conjunctival hyperemia that is more pronounced in the perilimbal area. Limbal involvement is marked by the presence of sticky mucous filaments, gelatinous limbal infiltrates with Horner-Trantas dots, and modest superficial paralimbal punctate keratitis in the upper sector of the cornea. Slit-lamp examination of the girl's left eye (**B**, lower panel) only showed a slight presence of diffuse conjunctival hyperemia.

At the eye examination, visual acuity resulted in 20/20 in both eyes, with cycloplegic refraction of mild hyperopia: +1.00 sph. A slit-lamp examination of her right eye revealed that she had a moderate inflammation of the bulbar conjunctiva, which was more pronounced in the perilimbal area. It was also possible to notice the presence of sticky mucous filaments and gelatinous limbal infiltrates with Horner-Trantas dots, particularly in the superior and lateral areas. Additionally, the cornea had a modest superficial paralimbal punctate keratitis in the upper sector (Figure 1). At the eversion of the upper lid, there were no giant papillae of the tarsal conjunctiva. In contrast, the left eye was normal upon examination, with only a slight presence of diffuse conjunctival hyperemia and with no evidence of punctate keratopathy. From a general point of view, apart from allergic asthma, the patient had no other health issues and she was not affected by atopic dermatitis. Given the clinical course and the ocular signs, a diagnosis of unilateral limbal VKC was made. The girl was treated with 1% cyclosporine eye drops to be applied four times a day on the right eye. At the one and three-month follow-ups, the ocular symptoms had significantly improved, and no side effects, including allergic reactions, were observed.

**Figure 2:**
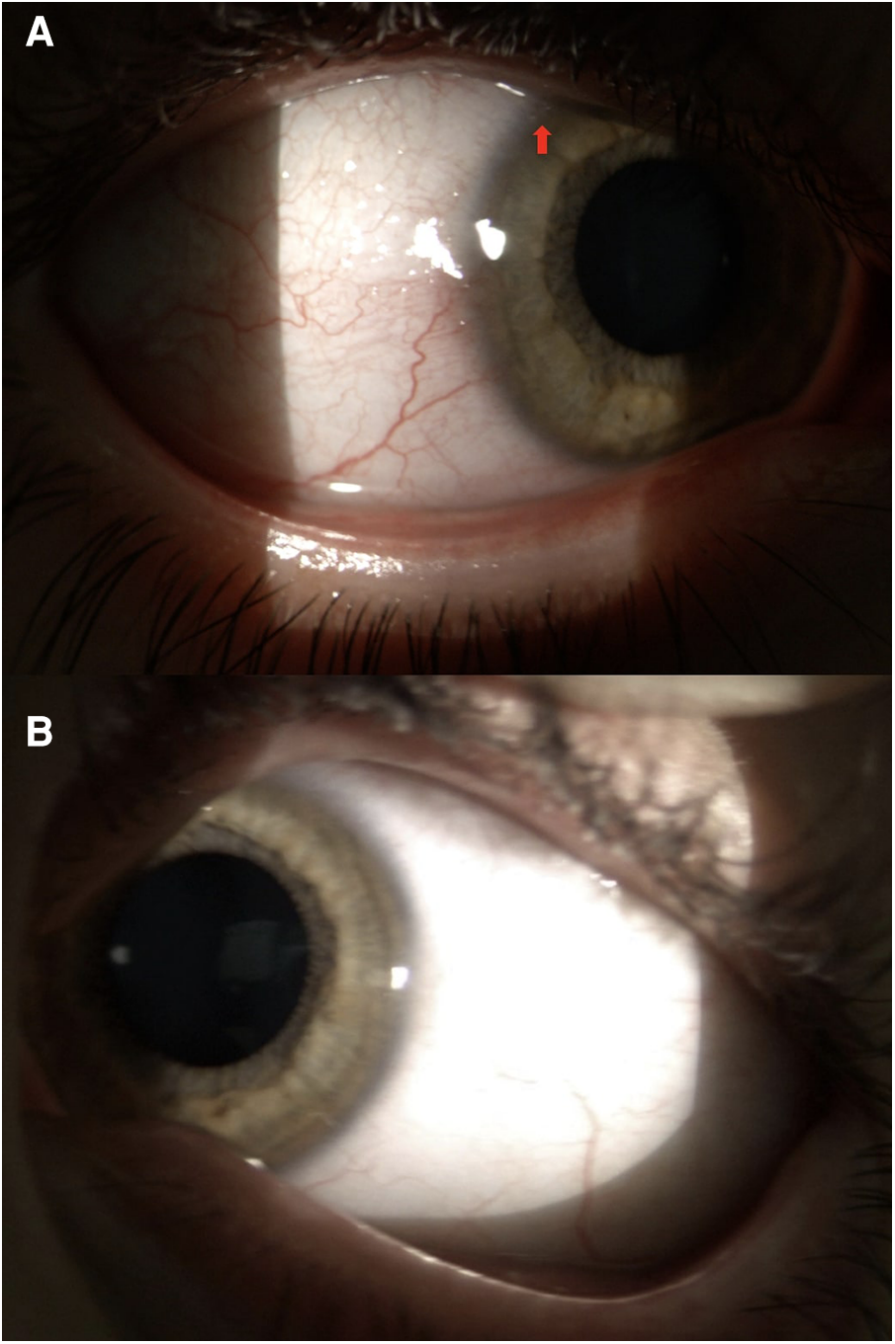
Slit-lamp examination of the 7-year-old boy's right eye (**A**, upper panel) shows a unilateral limbal VKC. Small round limbal nodules (papillae) and two small Horner-Trantas nodules (arrow) can be noticed in the superior and lateral regions. Eyelid margins are not involved. The boy's left eye (**B**, lower panel) was normal upon examination.

We also examined the patient's 7-year-old brother. His history was not remarkable for atopic dermatitis, neither allergic asthma nor rhinitis. Prick tests conducted by an experienced pediatric allergist to evaluate allergies related to dust mites, birch trees, grass pollen, mugwort, oleaceae, parietarian, dog hair, and cat hair, indicated the absence of IgE-mediated allergies. He also did not have any other underlying medical conditions. However, he did have a red eye, which had been present for a few weeks at the time of the visit, and unilaterally affected his right eye. According to the parents, he had been rubbing his right eye for a few months before the redness appeared. This symptomatology coincided with the onset of warmer weather in springtime and worsened notably at the beginning of summer. There was no runny nose or nasal itching. No treatment had been carried out at the time of the examination. He had normal visual acuity of 20/20 and a refraction of +0.75 sph in both eyes. Upon thorough examination, the patient's right eye revealed signs of conjunctival hyperemia with small round limbal nodules (papillae) of modest size, located superiorly and laterally. Two of these (on the upper limbus) had small Horner-Trantas nodules at their apex, typical of the active phase of the disease (Figure 2). There was no evidence of involvement of the eyelid margins, indicating the absence of blepharitis (and subsequent blepharoconjunctivitis). Based on these findings and also on the typical onset age and on the male sex of the patient, a diagnosis of unilateral limbal VKC was established in the patient's right eye, albeit in a milder form compared to that of his sibling. We also found a conjunctival hyperemia in the left eye, with no evidence of punctate keratopathy in both eyes, examined after the application of fluorescein drops. The boy was treated with hydrocortisone eye drops, to be applied on the right eye twice a day for 7 days. This treatment was repeated a second time within the following three months, due to the resuming of itching. No side effects were observed. At the three-month follow-up, the ocular condition remained substantially unchanged.

**Table:** Timeline showing the key stages in diagnosing and treating the two patients.

Patient 1	
Event	Timeframe
Onset of conjunctivitis episodes in the right eye. Treatment with repeated 7−10 days cycles of tobramycin/dexamethasone eye drops with relapses at treatment discontinuation	5 years before first visit
Diagnosis of unilateral limbal VKC. Starting of treatment with 1% cyclosporine eye drops four times a day	First visit
Significant clinical improvements	1-month and 3-month follow-ups

**Table:** 

Patient 2	
Event	Timeframe
Onset of conjunctivitis symptoms	A few months before the first visit
Diagnosis of unilateral limbal VKC. Starting of treatment with a cycle of hydrocortisone eye drops twice a day for 7 days (repeated in the following months for resuming of the itching)	First visit
No significant changes in the ocular condition	3-month follow-up

## Discussion

To our knowledge, these are the first two reported cases of unilateral limbal VKC.

As regards the case of the girl, her clinical history and the typical clinical signs (3) led to a diagnosis of unilateral VKC, but the limbal form of the condition has never been previously reported in cases with unilateral eye involvement. In fact, the unilateral form of VKC has been previously reported only in the tarsal form, characterized by giant papillae in the tarsal conjunctiva (2, 5–7), and it occurs in only a few cases, ranging from 2% (2) to 3.3% (5). The association of asthma with VKC is reported in 14.6% of cases (5). While the pathogenesis of the disease is attributed to an IgE-mediated hypersensitivity reaction (4), this theory does not fully account for the possible unilateral localization of VKC.

On the other hand, the boy presented the disease at its onset, which is consistent with the literature indicating that VKC is more common in males and typically occurs between the ages of 6 and 7 (8). During the examination, we found that he also had unilateral limbal VKC, but unlike his sister, it was not attributable to allergic pathogenesis. It is known that approximately 50% of VKC patients have a negative allergy test, which suggests that non-IgE mechanisms may contribute to the inflammatory reaction in VKC (4). Due to the early stage of the boy's disease, it is difficult to predict its progression with certainty or determine whether his left eye will also be affected. However, the complete absence of symptoms and ocular signs in the left eye suggests a unilateral form of the disease.

These two cases of VKC exclusively affecting the right eye, in two individuals within the same family, provide evidence supporting a potential genetic hypothesis. Current knowledge about VKC is limited and previous studies have suggested that both genetic and environmental factors may contribute to the pathogenesis of VKC, given the demographic and geographical characteristics of the disease (4). Further studies are therefore needed to explain the occurrence of unilateral VKC cases.

While a Vernal-related unilateral eye involvement is very rare, these cases remind pediatric ophthalmologists and pediatricians that unilateral limbal Vernal keratoconjunctivitis can occur.

## Data Availability

The raw data supporting the conclusions of this article will be made available by the authors, without undue reservation.
